# The Role of Long Non-coding RNAs in Immunotherapy Resistance

**DOI:** 10.3389/fonc.2019.01292

**Published:** 2019-11-28

**Authors:** Yuwen Zhou, Yajuan Zhu, Yao Xie, Xuelei Ma

**Affiliations:** ^1^State Key Laboratory of Biotherapy, Department of Biotherapy and Cancer Center, West China Hospital, Sichuan University, Chengdu, China; ^2^Department of Dermatovenerology, West China Hospital, Sichuan University, Chengdu, China

**Keywords:** long non-coding RNAs, immune checkpoint inhibitor, immune response, immune evasion, immunotherapy resistance

## Abstract

T-cell-based immunotherapies, particularly immune checkpoint inhibitors, are promising treatments for various cancers. However, a large subset of patients develop primary or secondary resistance upon treatment. Although the detailed mechanisms remain unclear, immune escape via alterations in both cancer and tumor microenvironment has been identified as critical causes of immune resistance. Moreover, some long non-coding RNAs (lncRNAs), named as immune-related lncRNAs, have been recognized as regulators of immune cell-specific gene expression that mediate immune processes. These immune-related lncRNAs may play a vital role in immunotherapy resistance. Herein, we summarize current immune-related lncRNAs and their underlying roles in immune resistance to provide strategies for future research and therapeutic alternatives to overcome immunotherapy resistance.

## Introduction

Immunotherapies, particularly immune checkpoint inhibitors (ICIs), have gained attention worldwide because of their potential in disease treatment (1). The most representative ICIs, including programmed death (PD)-1, PD ligand (PD-L)1, and cytotoxic T lymphocyte-associated antigen (CTLA)-4, have been approved for treating various cancers (2, 3). Despite the unprecedented durable response rates of immunotherapies, these responses only occur in a subset of patients at a relatively low rate, ranging from 15 to 40% depending on the cancer type (4, 5). Most patients do not respond to these inhibitors because of primary or acquired resistance. Both primary resistance and acquired resistance result from complex and constantly evolving interactions between cancer and the immune system (6). Many mechanisms of immunotherapy resistance have been shown to be associated with immune evasion, showing (1) abrogated expression of cancer antigens; (2) decreased antigen presentation secondary to major histocompatibility complex (MHC), β2-microglobulin alterations; (3). overexpressed immune checkpoints (ICs) or their ligands, such as PD-1/PD-L1 and CTLA-4, T- cell immunoglobulin mucin domain-3 protein (TIM-3), and lymphocyte- activation gene 3; (4) severe exhaustion of T cells; and (5) increased activation and recruitment of immunosuppressive cells, such as tumor-associated macrophages, regulatory T cells (Tregs), and myeloid-derived suppressor cells (MDSCs) (7).

The human genome project and next-generation sequencing technologies revealed that only very small amounts of the genome encode proteins in mammal, while more than 90% of the genome is transcribed into tens of thousands of non-coding RNAs (ncRNAs) (8). Of these ncRNAs, long non-coding RNA (lncRNA) displays the most diversity and complexity and has various functions. The functions of some lncRNAs have been determined. LncRNAs control epigenetic modification and transcriptional regulation, by which they can regulate multiple biological activities, such as cell differentiation and development (9), cell cycle (10), and metabolic balance (11). Notably, accumulating evidence demonstrated that lncRNAs can modulate the immune response by controlling the homeostasis and functions of immune cells and anti- inflammatory factors (12). Therefore, these molecules are named immune-related lncRNAs (13). Given that their essential effects on the immune response are also associated with the causes of immunotherapy resistance (7, 13), lncRNAs may participate in immunotherapy resistance. Here, we review the potential role of lncRNAs in immunotherapy resistance to provide insight into the mechanisms of immunotherapy resistance from a new perspective.

## LncRNAs

An lncRNA is generally defined as an RNA with a molecule more than 200 nucleotides in length but without the ability to translate into protein (8, 14). It is the transcriptional product of RNA polymerase II and has a similar structure to mRNA but lacks an open reading coding frame. It is mainly distributed in the nucleus and cytoplasm. According to the localization of lncRNAs and length between protein-coding target mRNAs, lncRNAs can be approximately divided into intronic lncRNAs, long intergenic ncRNAs, antisense lncRNAs, transcribed pseudogene lncRNAs, and enhancer RNAs (13). Thousands of lncRNAs encoded within the human genome were initially considered as “transcription noise” or “dark matter” of genome without biological functions (15). However, with the application and development of high-throughput sequencing technology, an increasing number of lncRNAs with rich biological functions have been identified and named after their specific functions in various organisms. LncRNAs perform their functions by binding to DNA/RNA or proteins. Unlike mRNAs, lncRNAs do not have universal action modes of regulating gene expression and protein synthesis in different ways. LncRNAs are involved in the basic process of gene regulation at the epigenetic level, including chromatin modification, direct transcriptional regulation, and post-transcriptional functions, such as splicing, editing, localization, translation, and degradation (16). These multiple gene regulatory effects of lncRNAs have attracted extensive attention in cancer, and numerous studies have clarified that many lncRNAs are dysregulated in different cancers and play prominent roles in promoting and maintaining cancer hallmarks, such as proliferation, angiogenesis, apoptosis, and metastasis (17, 18). Several studies have indicated that lncRNAs not only involve the typical hallmarks of cancer but also are closely correlated with the regulation of cancer immunity by modulating the immune response (19).

## LncRNA Functions as an Immunomodulator

Some well-studied immune-related lncRNAs are reported to play a regulatory role in immune processes at an epigenetic level. In the innate immune response, lncRNAs modulate the production of inflammatory cytokines and functions of innate immune cells. Lethe, a pseudogene lncRNA, is a negative feedback regulator of the tumor necrosis factor α inflammatory signaling. It can bind to RelA, a subunit of nuclear factor (NF)-κB, to suppress the RelA-DNA binding, thereby inhibiting the expression and release of multiple inflammatory factors, such as interleukin (IL)-6 and IL-8 (20). Importantly, lncRNAs mediate both activation and repression of immune response genes. The lncRNA NeST (nettoie Salmonella pas Theiler's) is required for interferon (IFN)-γ synthesis in CD8+ T cells. It binds to WD repeat domain 5 (WDR5) to regulate histone methylation and expression of IFN-γ, therefore improving its antivirus and antibacterial effects (21). Similarly, lnc-DC, which is solely expressed in dendritic cells (DCs), is required for DC differentiation. In contrast, silencing of lnc-DC by stimulating the phosphorylation of signal transducer and activator of transcription 3 (STAT3) inhibits monocyte differentiation into DCs and reduces stimulation of T cells via DCs (22). In the adaptive immune response, lncRNAs modulate the differentiation and activation of T and B cells. Previous microarray analysis of CD8+ T cells revealed hundreds of differentially expressed lncRNAs involved in CD8+ T-cell activation and development of CD8+ memory and effector T cells (23). In B cells, lncRNAs can modulate the plurality, variety, and joining [V(D)J] recombination that are essential for generating antigen receptors (Ig or TCR) on antibodies (24). These findings strongly support that lncRNAs are critical immunomodulators and thus warrant further studies.

## LncRNAs and Immunotherapy Resistance

One of the central roles of the immune system is the surveillance and elimination of malignant transformations (25). However, malignant cells can escape from the immunosurveillance via diverse mechanisms, including reducing antigenicity to avoid attack by lymphocytes, inhibiting T-cell activity, and inhibiting immune response via upregulation of immunosuppressive factors and accumulation of immunosuppressive cells (26). Reactivating the immune system to the optimal is the key concept of cancer immunotherapy (27). As mentioned above, several studies suggest that lncRNAs could differentially regulate the T-cell-mediated immune response, resulting in the immunosuppressive environment, which may be significantly responsible for the immunotherapy resistance (Table 1). The lncRNAs may have therapeutic potential for overcoming immunotherapy resistance as an immune sensitizer. This implication is supported by the study of nuclear-enriched autosomal transcript1 (NEAT1). In the pre-clinical model, NEAT1 inhibition can attenuate CD8+ T-cell apoptosis and increases the cytolytic activity via the miR-155/Tim-3 pathway, enhancing the immune activity (35). NEAT1 is associated with the immunosuppression. The regression of NEAT1 implies an effective target for improving the outcome of immunotherapy. To date, there are no systematic reviews and summary about the relationship between lncRNAs and immunotherapy resistance. Therefore, it is essential to comprehensively describe these studies to obtain a better understanding of the role of lncRNAs in immunotherapy resistance.

**Table 1:** Overview of lncRNA function(s) implicated in the immunotherapy resistance.

**LncRNA** **name**	**Cancer type**	**Function(s)**	**Immune** **results**	**References**
LINK-A	TNBC	Induces antigen loss including PLC components (TPSN, TAP1, TAP2, and CALR) β2-microglobuli(β2M) and MHC class I and decrease CD8+ T cells and APC infiltration	–	(14)
meloe	Melanoma	Produces the most immunogenic MELOE antigens(MELOE-1, MELOE-2, and MELOE-3)	+	(28)
HOTAIR	Gastric/Cervical cancer	Promotes human leukocyte antigen (HLA)-G expression via inhibiting miR-152, miR-148a, respectively	+	(29, 30)
NKX2-1- AS1	Lung adenocarcinomas	Downregulates CD274/PD-L1	+	(31)
LINC00473	Pancreatic cancer	Upregulates PD-L1 and thus inhibiting activation of CD8+ T cells	–	(32)
MALAT1	DLBCL	Upregulates PD-L1 expression via miR-195 and regulates CD8+ T cells	–	(33)
SNHG20	Esophageal squamous cell carcinoma	Promotes growth and metastasis via ATM- JAK - PD - L1 pathway	–	(34)
NEAT1	Hepatocellular carcinoma	Attunes activity and promotes apoptosis of CD8+ T cells via regulating miR-155/Tim-3	–	(35)
NKILA	Breast and lung carcinoma	Improves the sensitivity to (activation-induced cell death) AICD of tumor-specific CTLs and TH1 cells	–	(36)
lnc-sox5	Colorectal cancer	Reduces infiltration and cytotoxicity of CD3+CD8+T cells via IDO1	–	(37)
HOTAIR	Leukemia	Leads to decreased ratio of CD4+/CD8+ T-cell subsets	–	(38)
Olfr29-ps1	Melanoma	Promotes the differentiation and function of MDSCs via the m6A- modified Olfr29-ps1/miR-214-3p/MyD88 regulatory network	–	(39)
lnc-chop	Melanoma Lung carcinoma breast cancer	Promotes the function and differentiation of MDSC	–	(40)
lncRNA Pvt1	Lung carcinoma	lncRNA Pvt1 is expressed on G-MDSCs and regulates the impressive activity of G-MDSC	–	(41)
lnc-MC	–	Promotes the differentiation of monocyte/macrophage into THP-1 cells and CD34(+) HSPCs via miR-199a-5p	+	(42)
RUNXOR	Lung caner	Accelerates MDSC-mediated immunosuppression	–	(43)
HOTAIRM1	Acute promyelocytic leukemia	HOTAIRM1 expression showed a markable association with myeloid differentiation	+	(44)
lnc-Smad3	–	Suppresses iTregs polarization and inhibits T-cell autoimmunity	+	(45)
lnc-EGFR	Hepatocellular carcinoma	stimulates Tregs differentiation, suppresses CTL activity	–	(46)
SNHG1	Breast cancer	Inhibits the differentiation of Tregs by Promoting miR-448 expression and reducing IDO level	+	(47)
Linc- POU3F3	Gastric cancer	Elevates the distribution of Tregs resulting in increasing cell proliferation by recruiting TGF-beta	–	(48)
Lnc-INSR	Acute lymphoblastic leukemia	Promotes Treg distribution and decreases the percentage of cytotoxic T lymphocytes	–	(49)

*Immune results ± represents the lncRNA regulating the immune response positively and negatively, respectively*.

*LINK-A, long intergenic non-coding RNA for kinase activation; TNBS, triple-negative breast cancer; PLC, peptide-loading complex; HOTAIR, HOX transcript antisense intergenic RNA; MALAT1, metastasis-associated lung adenocarcinoma transcript; DLBCL, diffuse large B-cell lymphoma; SNHG20, small nucleolar RNA host gene 20; NEAT1, nuclear enriched autosomal transcript 1; NKILA, NF-κ B-interacting long non-coding RNA; IDO, indoleamine 2,3-dioxygenase; lnc-chop, long non-coding RNA C/EBPβ homologous protein; lncRNA Pvt1, long non-coding RNA plasmacytoma variant translocation 1; lnc-MC, long non-coding RNA MonoCyte; RUNXOR, RUNX1 overlapping RNA; HOTAIRM1, HOX transcript antisense intergenic RNA myeloid1; EGFR, epidermal growth factor receptor; SNHG1, small nucleolar RNA host gene 1; lncRNA, long non-coding RNA; INSR, lnc insulin receptor precursor*.

### LncRNAs Affect Antigen Presentation

Antigen presentation marks the initiation of the immune response. Normally, antigen-presenting cells (APCs) are required to take up and present cancer cell antigens with the help of major histocompatibility complex class I (MHC-I) molecules to the activated responding CD8+ T cells (50). However, in some cases, due to functional deficiency in proteasome members, transporters, MHC itself, or beta-2-microglobulin (β-2M) during antigen processing, antigen presentation is rendered ineffective. Of note, β-2M, one of the chains of MHC- I, plays a key role in the folding and transport of the human leukocyte antigen I (HLAI) family to the cell membrane. If β-2M function is aberrant, CD8+ T cells may lose the ability for recognition of cancer antigens, thus inducing immunotherapy resistance (51, 52).

In ~25% patients with triple-negative breast cancer (TNBC), high expression of long intergenic non-coding RNA for kinase activation (LINK-A) with low infiltration of APCs and activated CD8+ T cells is detected. It suggests that LINK-A negatively regulates the recruitment of APC and CD8+ T cells.

Furthermore, a decrease of β-2M and MHC-I expression is observed in patients with higher LINK-A expression. Mechanistically, LINK-A degrades TPSN, TAP1, TAP2, and CALR proteins of the peptide-loading complex (PLC), thus affecting the loading and editing of MHC-I. These findings suggest LINK-A may be a potential prognostic predictor, and using LINK-A inhibitors can enhance the effect of ICIs. Intriguingly, the treatment with LINK-A inhibitor (LINK-A LNA) increases the infiltration of hyperactivated CD8+ T cells in the tumor site rather than in the other tissues (14, 26, 53), while another lncRNA performs differently. The human leukocyte antigen-G (HLA-G), a member of the non-classical MHC family, inhibits the cancer immunity by abrogating NK cell activities (54). Expression of HOX transcript antisense intergenic RNA (HOTAIR) correlates positively with HLA-G expression in gastric cancer (GC). In the post-transcriptional regulation of miR-152, HOTAIR, as a competing endogenous RNA (ceRNA), upregulates HLA-G expression, thereby promoting immune evasion (29). Unexpectedly, lncRNAs can also improve antigen presentation after being translated into short polypeptides. In melanoma, lncRNA MELOE is translated into MELOE-1, MELOE-2, and MELOE-3 by different translational approaches. In addition, MELOE-1 shows the highest immunogenicity and can be recognized by tumor-infiltrating lymphocytes (TILs). It is currently considered a targeted specific antigen to improve the efficacy of melanoma immunotherapy (28).

### LncRNAs Regulate the PD-L1 Expression

Tumor-infiltrating CD8+ T cells express many inhibitory receptors, including PD- 1, B-, and T-lymphocyte attenuator, mucin domain-3 (TIM-3), lymphocyte-activation gene 3 protein (LAG-3), T-cell immunoglobulin domain, and the newly demonstrated T-cell immunoglobulin and immunoreceptor tyrosine-based inhibitory motif domain (TIGIT), to maintain the balance of immune response. Tumors also highly express certain ligands, such as ICs like PD-L1, which negatively regulate the immune response of antitumor T cells by binding PD-1, an inhibitory receptor on the cancer (26). The PD-1/PD-L1 axis is well-known to perform as a powerful IC—it limits T lymphocyte proliferation and toxicity effects, induces apoptosis of T cells, and promotes the differentiation of CD4+ T cells into Foxp3+ regulatory T cells and resistance of tumor cells to cytotoxic T-lymphocyte (CTL) attack (55). Furthermore, the overexpression of PD-L1 is associated with tumor progression and poor prognosis (56). High PD-L1 expression enables cancer cells to escape from the host immune system (55), thereby indirectly leading to immunotherapy resistance.

The lncRNA metastasis-associated lung adenocarcinoma transcript 1 (MALAT1), widely expressed in mammal tissue, was first identified in lung cancer. In diffuse large B-cell lymphoma (DLBCL), MALAT1 could sponge miR-195 to upregulate the expression of PD-L1, thus promoting migration and immune escape by regulating proliferation and apoptosis of CD8+ T cells. Inhibition of MALAT1 could rescue these events and attenuate the epithelial-mesenchymal transition-like process (33). Similarly, LINC00473 is highly induced in pancreatic cancer and is associated with poor outcome. It upregulates the expression of PD-L1 by sponging miR-195-5p in pancreatic cancer. Conversely, when LINC00473 is inhibited or miR-195-5p is upregulated, the consequently downregulated PD-L1 augments the enhanced CD8+ T cells, thereby suppressing the development of cancer (32). Additionally, small nucleolar RNA host gene 20 (SNHG20) increases the expression of PD-L1, ataxia telangiectasia mutated kinase (p-ATM), and p-JAK1/2 in esophageal squamous cell carcinoma. It serves as a carcinogen and promotes proliferation and metastasis via modulating the ATM/JAK- PD-L1 pathway (34). The gene CD274 has been identified to encode PD-L1. In lung cancer, lncRNAs NKX2-1-AS1 and NKX2-1 (also known as thyroid transcription factor 1, TTF-1) are coexpressed but work differently on the expression of CD274.

NKX2-1 protein can activate the transcription of CD274 by binding to its gene promoter. However, NKX2-1-AS1 disturbs this process to reduce the production of CD274 mRNA, resulting in the downregulation of PD-L1. Importantly, after the knockdown of NKX2-1-AS1, PD-L1 expression increases (31). Therefore, high expression of NKX2-1-AS1 is demonstrated as a favorable factor for the suppression of immune evasion by downregulating PD-L1.

### LncRNAs Modulate T Cells

Under normal physiological conditions, T cells recognize cancer cells, infiltrate at the tumor site, and exert cytotoxic effect, thereby killing the cancer cells. However, in advanced cancer, T cells display an exhausted or unresponsive state, in which their functions are impaired because of high tumor-antigen load and immunosuppressive factors in the tumor microenvironment (57). In mouse models of melanoma, tumor-inherent activation of WNT/β-catenin signaling pathway has been found to restrain T cells from populating the tumor and lead to T-cell exclusion, which in turn results in the primary resistance against PD-L1/CTLA-4 treatment (58). In a clinical study, non-small-cell lung carcinoma (NSCLC) patients treated with PD-1 blockade (pembrolizumab), the ones with more CD8+ T-cell infiltration have a durable response, whereas in advanced patients, CD8+ T cells are at a rejected state. The number of CD8+T cells has been confirmed to be a potent indicator of immunotherapy response (59). Therefore, infiltration of the weakened CD8+ T cells is commonly recognized as responsible for immunotherapy (23).

Binding of FasL (CD95L) and Fas (CD95) between T cell-B cells or T cell-T cells can initiate activation-induced cell death (AICD) to eliminate T cells or B cells, thus regressing immune response by exhausting T cells (58). LncRNA NKILA, known as nuclear factor-κB (NF-κB)-interacting lncRNA (NKILA), can improve the sensitivity of tumor-specific CTLs and type 1 helper T (TH1) cells, resulting in AICD by inhibiting NF-κ B activity (36). In the cases of breast and lung cancer, patients with high expression of NKILA in CTLs and TH1 cells reveal poor outcome (60). Moreover, knockdown of NKILA significantly inhibits tumor growth by increasing the CTLs in tumor (36). Lnc-sox5, previously reported to be ultra-highly expressed in tongue tumor (60), was found inhibiting T-cell activity. Inflammation can induce more indoleamine 2,3-dioxygenase 1 (IDO1), which benefits cancer immune escape by producing kynurenine. Kynurenine in the tumor environment can dampen the growth and function of T cells and NK cells (61). A significant increase of lnc-sox5 is observed in colorectal cancer. Knockdown of lnc-sox5 highly decreases the production of IDO1 and enhances the cytotoxicity of CD3+CD8+ CTLs at the tumor site (37). These findings indicate that lnc-sox5 can modulate the immune environment to promote tumor progression. NEAT1 has been confirmed to be a crucial oncogene in multiple types of cancer. Dysregulation of NEAT1 promotes the progression of cancer by accelerating proliferation, migration, and evasion (62, 63). Currently, it has been revealed that NEAT1 contributes to immune escape by restraining the antitumor function of T cells. Of note, T-cell immunoglobulin and mucin domain protein 3 (Tim-3) are upregulated in hepatic cell carcinoma. Tim-3 can induce CD8+ T-cell fatigue and participates in the death of CD8+ T cells. Downregulated NEAT1 suppresses CD8+ T-cell apoptosis and enhances cytolysis through the miR-155/Tim-3 pathway (35). Taken together, these reported lncRNAs exert an inhibitory effect on the activities of T cells, particularly CD8+ T cells. These lncRNAs may have a vital influence on immunotherapy and display the potential to be targets for elevating immunotherapy.

### LncRNAs Control the Recruitment and Activity of Immunosuppressive Cells

Some immune cells have been known as key roots of immune suppression. The presence of MDSCs and Tregs in the tumor microenvironment is associated with poor survival and low response rates to ICI therapy (64). Human MDSCs express CD11b and CD33 but not HLA-DR as well as the lineage- specific antigens CD3, CD19, and CD57 (65). MDSCs, consisting of myeloid progenitors, precursors of macrophages, granulocytes, and dendritic cells, are major immune regulators in a variety of pathological conditions, particularly in tumors (66). MDSCs release arginase-1 (Arg-1), NO synthase 2 (NOS2), NADPH oxidase 2 (NOX2), and cyclooxygenase-2 (COX2). They produce various toxic and regulatory substances such as H_2_O_2_, damage nucleic acids, proteins, and lipids, and produce reactive oxygen species (ROS), which attenuate T-cell activities (67). In addition, MDSCs diminish local nutrients, which are required for the T-cell expansion (68). On the other hand, MDSCs also promote the expansion of induced Treg (iTreg) cells and inhibit the response of natural killer T (NKT) cells (69). Based on the immune suppression of MDSCs, which contributes to the immune escape, inhibition or elimination of MDSCs is regarded as a feasible therapeutic strategy to enhance the immunotherapy (66). As such Tregs, characterized by expression of Forkhead box protein P3 (FoxP3), are divided into two distinct subsets: naive Tregs (CD4+CD45RA+FOXP^3*low*^) and effector Tregs (CD4+CD45RA–FOXP3^*high*^). The immature Tregs proliferate and differentiate into the effector Tregs upon the action such as reaching the tumor site. Effector Tregs are suggested to suppress antitumor immunity because they inhibit the effector T-cell response by secreting inhibitory cytokines, such as IL-10, IL-35, and TGF-β or by direct cell contact and also augment the infiltration and differentiation of immunosuppressive cells such as tumor-associated macrophages (TAMs) and cancer-associated fibroblasts (CAFs) (4, 70). Moreover, in the murine model, the removal of Tregs from the tumor microenvironment significantly elevates the immune effects (71). Overall, MDSCs and Tregs in the tumor environment are responsible for the immune therapy resistance.

Several lncRNAs are proven to regulate the recruitment and activity of immunosuppressive cells, such as MDSCs and Tregs. The pseudogene lncRNA Olfr29- ps1 is expressed in MDSCs and can promote MDSC differentiation into monocytic (Mo-) MDSCs with higher suppressive activities. LncRNA Olfr29-ps1 competitively binds and downregulates the miR-214-3p, thus upregulating the expression of its target gene *MyD88* to modulate the transformation of MDSCs. Additionally, the N6- methyladenosine (m6A) modification via IL6 is required to enhance Olfr29-ps1 expression and augment the binding of Olfr29-ps1 with miR- 214-3p (39). Likewise, Lnc-chop, a newly discovered lncRNA, controls the function and differentiation of MDSCs in tumor and inflammatory environments. Knockdown of lnc-chop in MDSCs increases the release of IFN-γ by the CD4+ and CD8+ T cells; however, the differentiation of more immunosuppressive M-MDSCs decreases. The regulatory mechanism has been elucidated as the following: the transcription factor CCAAT- enhancer-binding protein β (C/EBPβ) controls the gene expression of *Arg-1, NOS2, NOX2*, and *COX2*. Lnc-chop promotes the activation of C/EBPβ and upregulates the expression of *Arg-1, NOS2, NOX2*, and *COX2* via binding to both C/EBPβ homologous protein (CHOP) and liver-enriched inhibitory protein (LIP), thus inducing the immune suppressive environment. More importantly, lnc-chop increases the production of NO, H_2_O_2_, and ROS and the expression of *Arg-1* by promoting the enrichment of the histone H3 lysine 4 trimethylation (H3K4me3) in the promoter region of *Arg-1, NOS2, NOX2*, and *COX2* (40). Plasmacytoma variant translocation 1 (PVT1), an lncRNA encoded by the human *Pvt1* gene, is related to the regulation of granulocytic myeloid-derived suppressor cells (G- MDSCs). Under hypoxia, the hypoxia-inducible factor (HIF)-1α upregulates *Pvt1* expression in G-MDSCs. *Pvt1* plays a critical role in regulating the immunosuppressive functions of G-MDSCs. *Pvt1* silencing decreases the *Arg1* and ROS levels in G-MDSCs and restored antitumor T-cell responses (41). Therefore, the known immune-related lncRNAs mainly positively regulate the immunosuppressive abilities of MDSCs and contribute to the immune evasion, which potentially leads to immunotherapy resistance.

However, lncRNAs show strong dual effects on the differentiation and distribution of Tregs. Lnc-Smad3 and H3K4 methyltransferase Ash1l show opposite effects in polarization of Tregs by regulating the Foxp3 locus in an opposite manner. TGF-β activates Smad proteins, including Smad2 and Smad3, by phosphorylation, and then Smad complex binds to the Foxp3 locus, inducing its expression, which polarizes Treg cells. Ash1l has been known to directly target the *Smad3* promoter to increase the H3K4 trimethylation and then upregulate the Smad3 expression, while lnc-Smad3 restricts Smad3 transcription by interacting with histone deacetylase 1(HDAC1). When TGF-β is stimulated, activated Smad inhibits lnc-Smad3 to bind Ash1l, thus inducing iTreg polarization (45). On the contrary, lnc-epidermal growth factor receptor (EGFR) leads to immunosuppressive state to cancer. Mechanistically, lnc-EGFR can induce EGFR expression via binding to EGFR, thus promoting differentiation and distribution of Tregs (46). In pediatric acute lymphoblastic leukemia, Lnc-insulin receptor precursor (INSR) abnormally activated NSR and the phosphatidylinositide 3-kinase/AKT signaling pathway, enhancing Treg cell differentiation (49) and may offer valuable therapeutic targets in the immune suppression of tumor microenvironment.

## Summary

**Figure 1:**
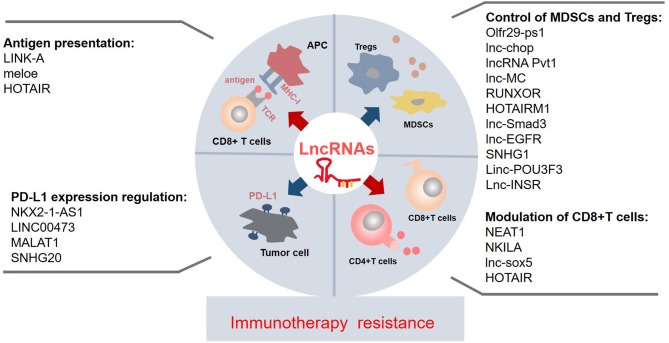
The known immune-related lncRNAs may play a vital role in the immunotherapy resistance via regulating the immune components and environment at different levels and by a myriad of mechanisms. The suppressive antigen presentation, upregulated PD-L1 expression on the tumor, the dysfunctions of T cells, and accumulation of immunosuppressive cells contribute to the immunotherapy resistance. A few lncRNAs affect the process of antigen presentation; they impair/enhance the MHC-I function or produce specific antigens. Some lncRNAs regulate the PD-L1 expression on the tumor, especially upregulating the PD-L1 expression. Majority of lncRNAs control the recruitment and activity of MDSCs and Tregs; most of them are upregulated in the MDSCs and Tregs. Collectively, most of known immune-related lncRNAs may contribute to the immunotherapy resistance. lncRNAs, long non-coding RNAs; Tregs, regulatory T cells; MDSCs, myeloid-derived suppressor cells; APC, antigen-presenting cell; PD-L1, programmed cell death protein 1; TCR, T-cell receptor; MHC-I, major histocompatibility complex.

Despite lncRNAs only recently becoming a hot topic of immense research interest, compelling evidences have revealed that lncRNAs have multiple functions as systemic regulators in biological processes. Regulation of immune-related lncRNAs may also play a crucial role in immunotherapy resistance. First, most reported immune-related lncRNAs contribute to immunotherapy resistance through inducing immune evasion at different stages, including the loss or weakness of antigen presentation, upregulation of PD-L1 expression, attenuation of T-cell activities, and accumulation or immunosuppressive capacity increase of G-MDSCs and Tregs in tumor environment (Figure 1). Second, since the advent of immune evasion is closely associated with cancer development and growth, NEAT1 may have oncogenic properties and induce tumor growth. In addition, the coexpression of lncRNAs and tumorigenesis gene (well-known oncogene *MYC* and lncRNA Pvt1) is found in various tumors (41), which suggests that lncRNAs may have an important direct relationship with tumor growth. Finally, lncRNAs play a critical role in immune evasion. For example, HOTAIR not only promotes HLA-G expression to enhance the antigen presentation but also downregulates CD4+/CD8+ T-cell subsets (Figure 1; Table 1) (29, 30, 38), suggesting the dual effects of lncRNAs, which should be considered in future studies. These findings of immune-related lncRNAs in cancer immune regulation may provide potential theoretical strategies or novel approaches to overcome immune resistance through the manipulation of lncRNAs. However, only a small population of immune-related lncRNAs are studied in individual cancer. In addition, the importance of an lncRNA may vary in different types of tumors. Future explorations are warranted to discover more immune-related lncRNAs and elucidate their common mechanisms of immune regulation in various types of cancers, thus giving more direct indications to deal with immune resistance.

## Author Contributions

YZhou collected and reviewed the literature and wrote the manuscript. YZhu wrote and revised the manuscript. YX rechecked the manuscript and put forward meaningful comments on it. XM contributed to writing design and revised the manuscript. All authors read and approved the final manuscript.

### Conflict of Interest

The authors declare that the research was conducted in the absence of any commercial or financial relationships that could be construed as a potential conflict of interest.
